# Experimental imaging and Monte Carlo modeling of ultrafast pulse propagation in thin scattering slabs

**DOI:** 10.1117/1.JBO.27.8.083020

**Published:** 2022-06-02

**Authors:** Lorenzo Pattelli, Giacomo Mazzamuto

**Affiliations:** a Istituto Nazionale di Ricerca Metrologica (INRiM), Torino, Italy; b European Laboratory for Nonlinear Spectroscopy (LENS), Sesto Fiorentino, Italy; c Istituto Nazionale di Ottica (INO), CNR, Sesto Fiorentino, Italy

**Keywords:** multiple scattering, thin layered media, semitransparent media, optical gating, time of flight spectroscopy

## Abstract

**Significance:**

Most radiative transport problems in turbid media are typically associated with mm or cm scales, leading to typical time scales in the range of hundreds of ps or more. In certain cases, however, much thinner layers can also be relevant, which can dramatically alter the overall transport properties of a scattering medium. Studying scattering in these thin layers requires ultrafast detection techniques and adaptations to the common Monte Carlo (MC) approach.

**Aim:**

We aim to discuss a few relevant aspects for the simulation of light transport in thin scattering membranes, and compare the obtained numerical results with experimental measurements based on an all-optical gating technique.

**Approach:**

A thin membrane with controlled scattering properties based on polymer-dispersed ${\mathrm{TiO}}_{2}$ nanoparticles is fabricated for experimental validation. Transmittance measurements are compared against a custom open-source MC implementation including specific pulse profiles for tightly focused femtosecond laser pulses.

**Results:**

Experimental transmittance data of ultrafast pulses through a thin scattering sample are compared with MC simulations in the spatiotemporal domain to retrieve its scattering properties. The results show good agreement also at short distances and time scales.

**Conclusions:**

When simulating light transport in scattering membranes with thicknesses in the orders of tens of micrometer, care has to be taken when describing the temporal, spatial, and divergence profiles of the source term, as well as the possible truncation of step length distributions, which could be introduced by simple strategies for the generation of random exponentially distributed variables.

## 1 Introduction

The Monte Carlo (MC) method for radiative transport is known as the gold standard for the description of light scattering in turbid media, especially when their optical density does not satisfy the assumptions of the diffusive approximation. The presence of weakly scattering or even optically clear regions inside a scattering medium can alter significantly the overall propagation of light, which is relevant for a number of cases including for instance, in the biomedical field, the thin cerebrospinal fluid layer,^1^ the vitreous humor,^2^ and optically cleared tissues in general.^3^

In all these cases, the reduced scattering mean free path $l_{s}^{\prime }$ of these materials is comparable to or even larger than the thickness of these layers, thus requiring an MC description of light propagation. Owing to the intrinsic rescalability of the MC trajectories and sample geometry,^4^^,^^5^ this typically holds true irrespective of the actual (absolute) length scales involved. In practice, however, scattering samples that are both optically thin and physically thin pose additional challenges that have been rarely considered in the literature. A part of these difficulties arise from the fact that the energy velocity of light remains fixed upon geometrical rescaling operations, and therefore the typical time scales associated with the radiative transport process can become extremely fast for minute specimens, making time-domain experiments more difficult to perform as they require ultrafast laser sources and, more importantly, ultrafast detection apparatuses with sub-ps resolution.

Nonetheless, studying light transport at these extreme time scales can bring valuable insights. For instance, the details of early sub-ps transients are associated with the single scattering regime even in diffusive media,^6^^–^^9^ reveal structural properties related to the heterogeneity of scattering samples^10^^,^^11^ and carry information about the scattering process down to the micrometer scale, which is also relevant for the study of natural and biological materials.^12^^–^^14^ Prominent examples include thin tissues or small bioptic samples,^15^^–^^18^ cell suspensions^19^ or optical tissues which are often in the submillimeter range,^20^^–^^23^ thus requiring femtosecond-scale pulsed excitation for their optical characterization.

As the micrometer-scale becomes relevant, analog considerations apply to the spatial domain too. While one can often consider a collimated laser beam as a reasonable representation of a pencil beam, this may not be valid anymore if the thickness of the scattering layer becomes comparable to the waist of the impinging beam. In a typical experiment on very small specimens, tightly focused beams are typically used, which however are associated with a strong divergence after a short Rayleigh length. Thus, the need for an accurate description of tightly focused beams is of particular relevance in these circumstances.

Finally, due to the fast transport dynamics in thin media, which is dominated by ballistic or quasiballistic propagation, large MC simulations are usually required to accumulate a sufficient statistics and reach the multiple scattering regime. Simulations comprising billions or even more trajectories are not infrequent in this case, which however require extra care to make sure that the generation of random variates follows the intended distributions and is not affected by finite precision truncation effects.

In this report, we discuss a few modifications that can be introduced into a standard MC implementation to mitigate the possible shortcomings related to ultrafast time-of-flight measurements. Simulation results are then compared with experimental data from an all-optical gating setup capable of recording the temporal evolution of the transmittance profile from a simple plane-parallel scattering slab at the transition between the ballistic and diffusive regime, showing good agreement with the numerical profiles.

## 2 Materials and Methods

### 2.1 Source Description

Light transport in optically thin samples is associated with several deviations from the simple diffusive behavior, which are particularly interesting to study in the time domain.^24^^–^^27^ In this context, MC simulations are successfully used to reproduce and explain the observed transport properties, especially when the samples have a macroscopic extent.

For much smaller sample sizes, much shorter time scales become relevant which require the development of radically different experimental setups and detection schemes needed for sub-ps resolution,^8^^,^^28^^,^^29^ which could explain why time-domain experiments in this regime are rarely reported.

Optical gating techniques offer a straightforward strategy to achieve the required resolution by all-optical means, typically exploiting the Kerr effect or a nonlinear frequency generation process. In either case, an ultrafast laser source is required for the fast gate pulse, which is typically obtained via broadband Ti:Sa laser sources. When operated in the femtosecond pulse regime, the onset of mode-locking via a combination of self-phase modulation and group velocity dispersion leads to the formation of stable pulses^30^ with a characteristic temporal profile

$$I(t;\Delta \tau_{p})=\sqrt{\frac{\gamma_{p}}{2}}{\mathrm{sech}}^{2}(\gamma_{p}t),$$ (1)

with $\gamma_{p}=2\;\ln(1+\sqrt{2})/\Delta \tau_{p}$ and $\Delta \tau_{p}$ being the full width at half-maximum of the pulse. This profile shape is characterized by exponentially decaying tails, which thus extend much further in time with respect to a Gaussian pulse profile. What can be typically measured is the autocorrelation of a ${\mathrm{sech}}^{2}$ pulse with itself, which still presents the same exponentially decaying tails and can still be expressed analytically as

$$I_{\mathrm{ac}}=\gamma_{\mathrm{ac}}{\mathrm{csch}}^{2}(\gamma_{\mathrm{ac}}t)[\gamma_{\mathrm{ac}}t\;\coth^{2}(\gamma_{\mathrm{ac}}t)-1],$$ (2)

with $\gamma_{\mathrm{ac}}=2\;\ln(1+\sqrt{2})/\Delta \tau_{\mathrm{ac}}$. Notably, the expected spectrum for a ${\mathrm{sech}}^{2}$ pulse, as obtained by a Fourier transform operation, is still given by a ${\mathrm{sech}}^{2}$ curve as

$$\overset{˜}{I}(\omega ;\Delta \tau_{p})=\frac{\pi^{2}}{2\gamma_{p}}[{\mathrm{sech}}^{2}(\frac{\pi^{2}}{2\gamma_{p}}(\omega -\omega_{0}))].$$ (3)

**Fig. 1:**
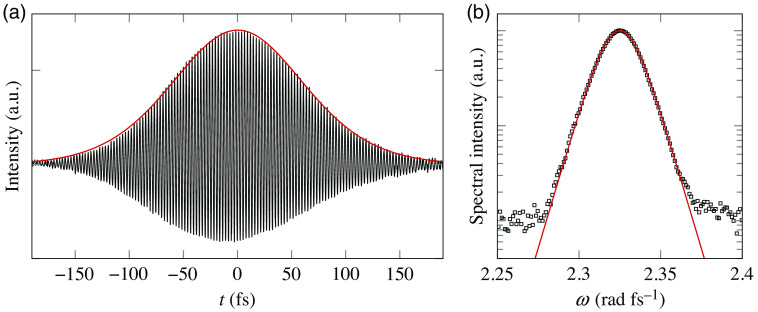
(a) Autocorrelation measurement of the Ti:Sa pulse. The envelope profile formed by the autocorrelations fringes is fitted with the autoconvolution of a ${\mathrm{sech}}^{2}(t)$ pulse, returning a full width at half maximum for the convoluted signal equal to 145 fs, corresponding to a pulse width of $\Delta \tau_{p}=94\;\mathrm{fs}$. (b) Spectrum of the Ti:Sa pulse centered at $\lambda_{0}=810\;\mathrm{nm}$, fitted with a ${\mathrm{sech}}^{2}(\omega -\omega_{0})$ function.

Figure 1 shows an example of temporal and spectral characterization of a ≈100  fs pulse from a Ti:Sa laser exhibiting the typical ${\mathrm{sech}}^{2}$ pulse and spectrum profiles. The output beam from this laser is then used to pump a passive parametric oscillator to generate a synchronous replica at a wavelength of 1510 nm, to be used interchangeably as the probe or gate pulse in the optical gating experiment.

The hyperbolic secant square distribution can be expressed as the logistic distribution

$$f(x;\mu ,s)=\frac{\exp(-\frac{x-\mu }{s})}{s(1+\exp(-\frac{x-\mu }{s}){)}^{2}}=\frac{1}{4s}{\mathrm{sech}}^{2}(\frac{x-\mu }{2s}),$$ (4)

which has a closed-form cumulative distribution function

$$F(x;\mu ,s)=\frac{1}{1+\exp(-\frac{x-\mu }{s})}=\frac{1}{2}+\frac{1}{2}\;\tanh(\frac{x-\mu }{2s}),$$ (5)

whose inverse or quantile function is straightforward to implement to generate random ${\mathrm{sech}}^{2}$ variates in an MC routine

$$Q(\xi ;\mu ,s)=\mu +s\;\ln(\frac{\xi }{1-\xi }),$$ (6)

with ξ uniformly distributed in the open interval (0, 1).

Experiments with small, submillimeter specimens typically require to focus the incoming beam tightly so that its beam waist is much smaller than the extent of the sample. Tightly focused beams are characterized by a strong divergence and a short Rayleigh length, which can easily become smaller or comparable to the thickness of the scattering layers. For instance, a beam focused down to a 10-μm spot size can have a Rayleigh length of ≈50  μm.

Due to its relevance, the accurate modeling of Gaussian beams has been addressed by several authors. Traditionally, trajectories are initialized to reproduce a certain focal distribution rather than an actual focal point.^31^ This approach has the advantage of being very easy to implement, even though it does not give the correct distribution of trajectories after or before the focal plane, which can be a critical factor when trying to evaluate accurately the deposition of energy in a tissue.

More advanced approaches have been proposed in recent years^32^^–^^34^ which can be however more or less suitable for certain applications, lead to infinite trajectory densities or be significantly more computationally intensive.

A particularly elegant approach that is able to correctly reproduce the profile of a Gaussian beam at every position was proposed by Milsom^35^ for the modeling of reverse saturable absorption. The approach is based on a stochastic ray-optics description which is perfectly compatible with an MC framework and based on the actual physical parameters used to describe the Gaussian beam.

**Fig. 2:**
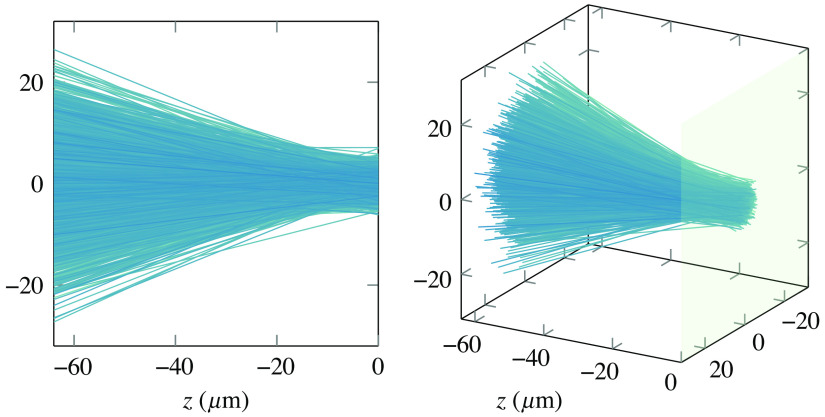
Views of a typical GRB focused in (0, 0, 0), with a beam waist of 8  μm in the focal plane. The envelope and intensity profiles formed by the rays follow the standard profile of a Gaussian beam at all z planes.

Briefly, following this approach each ray is defined in Cartesian coordinates as the trajectory connecting two points separated along z by a distance much larger than the Rayleigh length $z_{R}=\pi w_{0}^{2}/\lambda$, where $w_{0}$ is the spot size defined as the $1/e^{2}$ radius. A straightforward choice for the two z coordinates are at the focusing lens and at the beam focal plane. Assuming that the beam starts with a certain waist $w_{s}$ at a distance z=−d and arrives at the focal plane z=0 with a beam waist of $w_{0}$, then the pairs of points defining the rays in the bundle have coordinates $\left(x_{s},y_{s},-d\right)$ and $\left(x_{f},y_{f},0\right)$ given as

$$\begin{array}{cc} x_{s}=\frac{w_{s}}{2}{\mathrm{erf}}^{-1}(2\xi_{1}-1), & y_{s}==\frac{w_{s}}{2}{\mathrm{erf}}^{-1}(2\xi_{2}-1)\\ x_{f}=\frac{w_{f}}{2}{\mathrm{erf}}^{-1}(2\xi_{3}-1), & y_{f}=\frac{w_{f}}{2}{\mathrm{erf}}^{-1}(2\xi_{4}-1) \end{array},$$ (7)

where $\xi_{1\ldots 4}$ is four independent random numbers uniformly distributed in the open interval (0, 1). An illustrative representation of a resulting ray bundle is shown in Fig. 2 for a beam focusing at the entrance of a sample (z=0) with a beam waist of 8  μm.

Additionally, due to the ray-optics nature of the model, the so obtained Gaussian beam can be easily transformed using standard ray transfer matrix analysis when passing through interfaces, plates, lenses, and their combinations. The resulting bundle of rays will still follow the expected beam profiles as predicted by the theory.^35^

### 2.2 Numerical Aspects

MC techniques rely on the ability to sample random variables following well-defined probability distributions, which requires a good source of random numbers. For the purpose of computer simulations, pseudorandom number generators (PRNG) are sufficient as an efficient deterministic sequence of numbers characterized by an extremely long period, approximating the statistical properties of a truly random sequence. While the length of the PRNG periods and the memory footprint of their internal states do not represent a significant problem in modern implementations, care should be taken when considering how these floating-point random values are represented on a computer.

For instance, a 32-bit floating-point number can be represented with a precision of $2^{-149}$ near 0, but the precision drops to $2^{-24}$ over the [0.5,1) range. The finite and nonuniform precision of floating-point representation can have serious implications, especially for the generation of random numbers following probability density functions (PDFs) with infinite or semi-infinite support. In these cases, logarithms are often used to map values from a finite domain into an infinite range, such as when generating exponential variates for the step length

$$\ell =l_{s}\;\ln(1-\xi ).$$ (8)

Because there exists only a finite number of floating point representations, the tails are prematurely truncated, while the loss of precision introduces coarse gaps as the truncation value is approached. For the exponential distribution, in the common case of 32-bit PRNG, $-\ln\;2^{-32}\approx 22.18$, meaning that the probability of drawing a step length $\ell >22.18\times l_{s}$ is identically zero, instead of $2^{-32}$. Consequently, whenever a simulation requiring more than $2^{32}∼4\times 10^{9}$ steps needs to be run, it is possible that certain implementations of the step-length distribution based on Eq. (8) will hit the truncation limit and introduce a bias toward shorter step lengths. Using a 64-bit PRNG mitigates the problem by pushing the truncation farther, but more efficient and accurate solutions exist which should be preferred to avoid problems, especially when simulating time-resolved light transport in extremely thin films.

Truncation effects can be limited by developing custom PRNGs that are specifically designed to produce floating-point values.^36^^,^^37^ However, in the special case of the exponential distribution, truncation effects can be avoided entirely owing to its unique memorylessness property. This principle is exploited in the Ziggurat method,^38^ a rejection sampling algorithm that can be applied in general to monotonically decreasing PDFs. While MATLAB implements different versions of the Ziggurat algorithms since its first releases, other widely used libraries for scientific computing have implemented it only in recent years, such as the Boost C++ libraries (October 2016, version 1.62) or NumPy (July 2019, version 1.17). Notably, this extremely efficient rejection method is not well suited for GPU implementation, since each time one of the threads takes a branch into the “unlikely” rejection region, all other threads in the same warp must wait for it,^39^ introducing a critical performance penalty which can be (partially) mitigated by sacrificing a larger memory footprint.^40^

In the following, we will illustrate these aspects using a basic open-source MC code-named MCPlusPlus,^41^ which we have previously used for the simulation of scattering slabs with large statistics up to $\times 10^{14}$ trajectories,^29^^,^^42^ and which relies on the highly performing Boost Random library for the generation of exponentially distributed step lengths. If not otherwise specified, all simulations were performed using a focused ray bundle beam and a ${\mathrm{sech}}^{2}$ pulse profile, accumulating a statistics of $\times 10^{12}$ trajectories.

### 2.3 Sample Fabrication

Experimental time-domain measurements were performed on a scattering membrane with a low physical and optical thickness. The scattering sample is made of ${\mathrm{TiO}}_{2}$ nanoparticles (Huntsman Tioxide R-XL) dispersed in a UV-curable polymer matrix (Norland Optical adhesive 65). The opaque paste is stirred and sonicated before infiltration in a thin cell formed between two glass slides which were previously coated with water-soluble polyvinyl alcohol. The obtained sample is then cured under UV illumination and immersed in distilled water to detach it from the glass slides resulting in a free-standing scattering film with submillimeter thickness. As regards the scattering nanoparticles, we assume a refractive index of $n_{\text{rutile}}=2.5$ for the ${\mathrm{TiO}}_{2}$ and a particle diameter of ≈280  nm as declared by the manufacturer, which would correspond to an anisotropy factor of g=0.165. The nanoparticles should exhibit negligible absorption at the probe wavelength. Considering a volume fraction of 2%, we estimate an effective refractive index for the composite (${\mathrm{TiO}}_{2}+\text{polymer}$) material of $n_{\mathrm{MG}}=1.526$ using the Maxwell–Garnett approximation, which is well justified by the moderate index contrast and low volume fraction. This value is used as the effective refractive index of the scattering slab in the MC simulations. Based on the known particle/polymer volume fraction, the expected order of magnitude of the resulting transport mean free path is estimated between 40 and 50  μm based on Mie theory. Consequently, the thickness of the membrane was set to ≈160  μm to obtain a sample at the transition between the ballistic and the diffusive regime, using silica microspheres with a calibrated diameter as spacers between the glass slides. The optical properties of the optical adhesive are provided by the producer, with a nominal refractive index of $n_{\mathrm{pol}}=1.509$ at the probe wavelength of 1510 nm.

## 3 Results

The experimental measurements rely on an optical gating setup based on the collinear sum-frequency generation between a probe and a gate pulse impinging on a nonlinear crystal. This setup has been already used to characterize the time-resolved transmittance of different samples, including small specimens with a thickness of only a few micrometers, as described elsewhere,^9^^,^^43^ and has been recently expanded to provide wide-field time-resolved imaging to study light propagation both in scattering media^29^ and integrated photonic devices.^44^

**Fig. 3:**
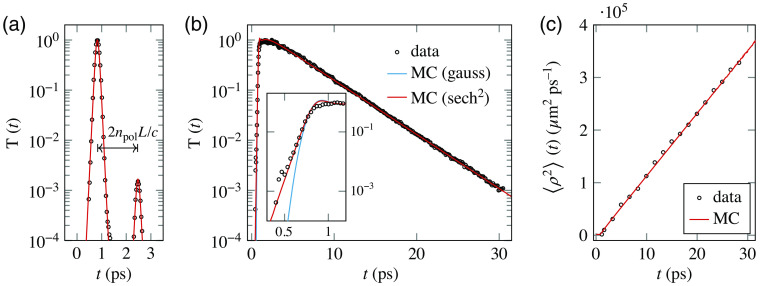
Time-domain measurements. (a) Experimental time trace relative to a transparent region of the sample, together with an MC simulation for a slab with $n_{\mathrm{pol}}=1.509$ and thickness L=164  μm. The ${\mathrm{sech}}^{2}$ pulse shape shows the same qualitative features of the experimental curve, however the experimental data results from the convolution between two ${\mathrm{sech}}^{2}$ pulses with different widths. (b) Comparison between a transmittance measurement and an MC simulation with a transport mean free path of 41.2  μm and an absorption coefficient of $\mu_{a}=4.5\times 10^{-5}\;\mu m^{-1}$, as determined by the mean square width growth data. The red and blue curves show simulation results obtained using a ${\mathrm{sech}}^{2}$ or a Gaussian pulse shape with equal full width at half-maximum. The difference between the two is only appreciable during the early transient (inset). (c) Mean square width data extracted from time-resolved images.

As a first measurement, the final thickness of the free-standing scattering slab can be estimated by performing a time-of-flight measurement through a transparent region of the sample, based on the time delay between the main transmitted peak and its first internally reflected replica. This measurement returned a thickness of L=164  μm, as shown in Fig. 3(a).

**Fig. 4:**
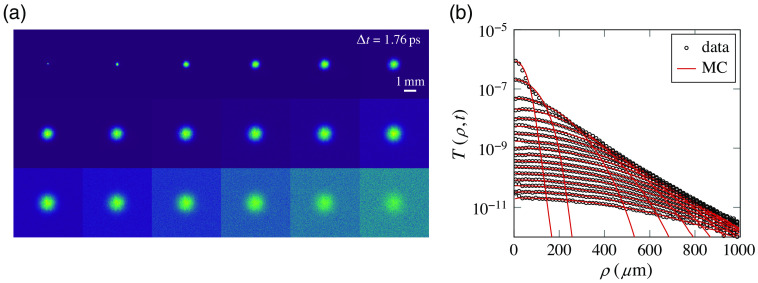
Time-resolved imaging measurements. (a) Set of acquired frames, each normalized to its maximum intensity. (b) Radial average of the spatial profiles for each frame, compared with MC data with $l_{s}^{\prime }=41.2\;\mu m$. Data points on the tails of early profiles are omitted for clarity. The amplitude of each curve is matched to that of the MC profile corresponding to the same delay.

A standard time-domain transmittance measurement taken with a photo-multiplier tube is shown in Fig. 3(b), together with the output of two MC simulations, showing the difference between a ${\mathrm{sech}}^{2}$ and a Gaussian pulse shape. While changing the pulse shape has a negligible effect in the multiple scattering regime (as expected), their difference is clearly visible during the early transient, which bears useful information on single scattering parameters.^9^ Our experimental setup also allows us to study the evolution of the spatial profiles at different delays using a CCD camera (Andor iKon M912). A useful parameter that can be extracted from these spatiotemporal profiles, and which we use in conjunction with the transmittance decay rate, is their associated mean square width calculated as

$$\langle \rho^{2}\rangle (t)=\frac{\int_{0}^{\infty }\rho^{2}T(\rho ,t)\rho \;d\rho }{\int_{0}^{\infty }T(\rho ,t)\rho \;d\rho },$$ (9)

which is shown in Fig. 3(c) for a collection of time-resolved frames. One advantage of working with the mean square width is that it is an inherently normalized figure which does not depend on the amplitude of the profile but only on its shape. For this reason, its values are exactly unaffected by the possible presence of absorption or other intensity variations (e.g., drifts or fluctuations of the laser source intensity). Therefore, one can directly infer the transport mean free path based on the growth rate of the mean square width. The value of the absorption coefficient, if any, can still then be evaluated in a second step by looking at the decay rate of the transmittance once the transport mean free path is known,^42^ taking it into account as a multiplicative factor. For our samples, we have found an experimental transmittance decay rate of 4.03(1) ps and a mean square width growth rate of $1.19(2)\times 10^{5}\;\mu m^{2}\;{\mathrm{ps}}^{-1}$. Compared with MC simulations, we find that these values correspond to a transport mean free path of 41.2(3)  μm and an absorption length of $\mu_{a}^{-1}=22(5)\;\mathrm{mm}$. The obtained transport mean free path is in good agreement with the Mie prediction based on the independent scattering approximation, despite the uncertainties in the true bulk density and refractive index of the nanoparticles, their polydisperse and nonspherical shape, and hence their actual volume fraction in the sample, which was estimated by their weight. On the other hand, even a small uncertainty on the transport mean free path leads to a much larger relative error on the absorption mean free path which is very sensitive to the decay rate of transmitted light and also more affected by the inherent noise in the experimental frames, which are shown in Fig. 4(a).

**Fig. 5:**
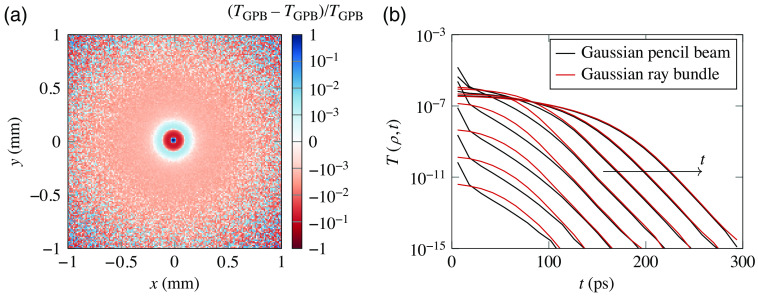
Numerical comparison between (a) spatial and (b) early spatiotemporal profiles obtained with a GPB or GRB source term focused on at the entrance of a 164-μm-thick sample with $l_{s}^{\prime }=41.2\;\mu m$. Especially at early times (spatiotemporal profiles cover a time span between 0 and 1.2 ps), the GRB source results in a more natural spatial spread of transmitted intensity due to its natural divergence.

The obtained spatiotemporal distributions are compared with those obtained by MC simulations in Fig. 4(b), showing good general agreement. The calibration of the spatial scale was characterized via transient imaging of a resolution target (not shown), returning a resolution of ≈13.5  μm/px. For the MC simulations, a ray-bundle beamwidth of 10  μm has been set based on a transient picture of the focal plane without the sample, which shows a single-pixel spot. The importance of using a Gaussian ray bundle (GRB) beam as opposed to a more common Gaussian pencil beam (GPB) spatial profile can be appreciated in Fig. 5. Therein, it can be seen that even for a sample with a thickness that is 4× the transport mean free path, a residual difference between the two models is still appreciable in transmittance as a ringing effect due to the finite divergence of the GRB model. This difference is dominated by the early transient where the spatiotemporal profiles are most different. As can be seen, the GRB model shows a more gentle spatial profile as opposed to the sharp ballistic peak of the GPB case, even though the two models converge to very similar profile shapes after a short transient of ≈1  ps.

## 4 Discussion

In this paper, we presented an MC adaptation of experimental data measured in the spatial and temporal domains on thin scattering films with a transmittance decay rate of the order of few picoseconds. This requires the use of tightly focused, ultrashort (i.e., sub-ps) laser pulses, which we have also modeled in a simple MC implementation inspired by MCML. Due to the fast decay rate, and the need to gather enough statistics to represent spatial profiles at different delays, MC simulations must be run with a large number of trajectories. When generating random numbers required to gather such a large statistics, care should be taken to avoid hitting the limitations of simple PRNGs and the direct implementation of quantile functions involving logarithms, as these could be associated with undesired truncation effects due to the finite precision of floating-point representations.

We should note that, in recent years, several alternative strategies to the accurate but computationally expensive MC approach have also been proposed to improve the description of transient light transport in small specimens. For instance, some authors suggested introducing an effective frequency-dependent diffusion coefficient,^26^^,^^45^ heuristic definitions for the boundary conditions^46^ or crossover lengths between the ballistic and diffusive regimes.^47^ Additionally, ongoing progress is being made with a number of analytical and semi-analytical methods to solve the radiative transfer equation (RTE) in the time domain, some of which can also be applied to model the transmittance through bounded geometries. Efficient $P_{3}$ solutions exist for the time-domain transmittance through a slab geometry,^48^ while other solutions are being developed following the rotated reference frame method, which however are used to study either the spatial domain or semi-infinite geometries^49^^,^^50^ and may still exhibit small deviations for samples with $L\approx l_{s}^{\prime }$. Computationally efficient solutions have been also reported using a colocated radial basis function method,^51^ the pseudospectral method,^52^ or within the framework of the broad class discrete ordinate methods,^53^ e.g., by combining it with spectral methods and Laplace transforms,^54^ convection schemes,^55^ finite-volume,^56^ or Fourier continuation methods.^57^ Finally, a promising approach has been recently proposed based on a dynamic radiative transfer system,^58^ which should be in principle capable of modeling light transport down to the femtosecond scale. However, to the best of our knowledge, none of these methods has been tested against experimental transmittance data on such short time scales.

Indeed, experimental studies on transient transport optically thin layers are still sporadic in the literature, despite the existing theoretical and numerical evidence that light transport in this geometry exhibits intriguing and counterintuitive properties.^29^^,^^59^ Given the increased availability of compact femtosecond-pulsed laser sources offering turn-key operation simplicity, optical gating detection schemes may become more widespread and bridge the gap toward the time and spatial scales that are relevant for submillimeter samples. Notably, all-optical gating techniques can typically provide a temporal resolution that is at least one order of magnitude higher than streak cameras, while providing wide-field (2D) imaging capabilities. Moreover, the probe-gate illumination scheme typically allows to enhance the upconverted signal by just increasing the intensity on the gate beam, and is therefore compatible with the study of delicate tissues and samples.

Robust and efficient methods to generate and handle large MC simulations will be increasingly needed to support the experimental investigations in this range of samples and geometries, as well as to validate the modeling approaches being developed for the description of early transients. At the sub-ps scale, the density of trajectories is potentially spanning over huge dynamic ranges,^58^ requiring the accurate simulation of extremely rare events such as exceedingly long steps tens of times larger than the average mean free path which may eventually dominate the transport properties at very large delays. The contribution of such extremely rare events remains likely negligible for most practical purposes: however, it could be interesting to check whether these extreme outliers arising naturally in semi-transparent media are compatible and/or necessary for the verification of the invariance property for the average pathlength under Lambertian illumination, which should in principle remain valid to an arbitrary degree of precision.^60^^,^^61^ Not least, on a more fundamental level, studying this extreme transport regime could provide insights regarding how far we can push the analogy between the scattering of light waves and the random walk model, on which all MC implementations implicitly rely.

## Code, Data, and Materials Availability

The data used for this study are available upon request. The Monte Carlo code is available at https://github.com/gmazzamuto/mcplusplus.
